# Understanding dog cognition by functional magnetic resonance imaging

**DOI:** 10.3758/s13420-017-0261-6

**Published:** 2017-02-25

**Authors:** Ludwig Huber, Claus Lamm

**Affiliations:** 1 Comparative Cognition, Messerli Research Institute, University of Veterinary Medicine Vienna, Medical University of Vienna, University of Vienna, Veterinaerplatz 1, A- 1210 Vienna, Austria; 2 Social, Cognitive and Affective Neuroscience Unit, Department of Basic Psychological Research and Research Methods, Faculty of Psychology, University of Vienna, Liebiggasse 5, A-1010 Vienna, Austria

**Keywords:** Dog, fMRI, Canine brain

## Abstract

Recent pioneering work has shown the great promise that scanning awake, nonsedated dogs holds for both understanding the canine and the human brain and mind. A number of technological and methodological challenges, however, still need to be overcome to fully tap this potential.

For both practical and theoretical reasons, one of the most highly investigated species in comparative psychology is the domestic dog (*Canis familiaris*). They are available in great numbers, but more importantly, they show surprisingly complex socio-cognitive skills, such as understanding heterospecific (human) gestures and discriminating facial expressions, which enable dogs to outperform even our closest primate relatives (e.g., Huber 2016). Pioneers of canine cognition research have thus proposed that the social abilities of dogs are functional matches of corresponding human abilities.

The reasons for the similarities between dogs and humans are manifold. Dogs, as a species, have a history of several thousand years alongside humans, and as individuals are raised in and live with human families. They have a common ancestry with wolves (*Canis lupus*), whose dependency on close cooperation with conspecifics, for breeding and hunting, presumably created similar selection pressures on their motivational and cognitive processes as on our human ancestors. This likely provided a good basis for the evolution of dog–human communication and cooperation.

Dogs have been tested not only for key abilities of human social cognition, such as imitation and perspective taking, but also for their understanding of human gestures, expressions, and even voice. But behavioral data alone are insufficient to determine whether similar behaviors across species can be explained by the same proximate mechanisms. With the advent of advanced, noninvasive imaging procedures, brain function related to the dog–human relationship and interspecies understanding may now be studied and compared in vivo, in both species. This, however, requires dogs to lie motionless in a noisy and shaky MRI scanner without anesthesia or sedation, as the latter would negatively affect both brain function and cognition. A breakthrough in this respect was achieved only a few years ago.

Four independent research groups—two in the USA (Atlanta and Auburn), one in Mexico (Querétaro), and one in Europe (Budapest)—have captured images of nonsedated and largely unrestrained dog brains, and their work and publications indicate the interest in and importance of this new frontier in functional neuroimaging (see Berns & Cook, 2016; Thompkins, Deshpande, Waggoner, & Katz, 2016, for review). Starting with investigations of reward processing, a few studies looked at perceptual (olfactory, auditory, and visual) processing, and very recently researchers examined executive functions (inhibitory control) and social and communication processes in the dog’s brain. Concerning the last domain, a group of canine researchers teamed up with neurobiologists specifically to test dog’s understanding of human speech. First, they found functionally analogous voice-sensitive cortical regions in the dog and human brain with a similar sensitivity to vocal emotional valence cues (Andics, Gacsi, Farago, Kis, & Miklosi, 2014). More recently, they also investigated how dog brains segregate and integrate lexical and intonational information from human speech (Andics, Gabor, Gacsi, Farago, Szabo, & Miklosi, 2016). That study suggests that brain activations in dogs processing linguistic cues are similar to those observed in humans, as indicated by differences in hemispheric lateralization, as well as the fact that praise words activated areas associated with reward processing. The latter two studies are particularly exciting as investigating how a nonhuman species responds to linguistic cues enables unprecedented insights into the origins of a skill thought to be uniquely human. Thus, they highlight the great potential that dog functional MRI (fMRI) offers, from a comparative perspective.

## Challenges and limitations

Dog fMRI clearly has the potential to result in major breakthroughs in understanding the sensory and cognitive processes of dogs. However, the evidence presented so far necessarily remains preliminary, because of its pioneering character and a number of methodological challenges that still need to be overcome. These are briefly described below in order to clarify that there are still ways to go before canine fMRI is comparable to human fMRI. This lag is based on the fact that so far all published studies have relied on measurement protocols and hardware developed for human imaging.

## Image quality

In comparison to the quality achieved with humans, the image quality in canine fMRI is rather low. This is mainly because radio frequency (RF) coils and imaging protocols have not been tailored to the specifics of dogs and their neuroanatomy. The dog’s brain is maximally one third the size of the human brain, with some critical brain areas deviating largely from the human counterparts, and its physiology (respiration rate, etc.) differs. We know from human neuroimaging that the coil’s performance must have excellent sensitivity and parallel imaging capability to enable the spatial and temporal resolution envisioned in contemporary canine neuroimaging. Published studies used different types of human MRI coils, none of which were designed for dogs. In particular, the coil geometries were not optimized for the canine head, and the number of RF channels close to the dog’s brain was limited. Because of the small number of channels, the expected imaging performance in terms of both temporal and spatial resolution is rather low compared to what can be achieved in human fMRI. This calls for the development of dedicated MRI coils, which in other species (e.g., in marmosets), has already shown great benefits.

Furthermore, the number of datasets acquired during a scanning session is limited by the short length of time dogs can restrain themselves. The limitations are less critical if simple conditioning and sensory processes are investigated. But it compromises the tremendous impact that more developed canine brain imaging could have for models of affect and cognition and for understanding the mechanisms of human social behavior. Thus, future training regimes should aim for longer scanning times, or make more extensive use of experimental protocols and analysis approaches which allow data to be aggregated over repeated imaging sessions with the same dog participants.

## Movement

A critical issue for achieving high-quality fMRI data in awake dogs is movement. Even the best trained dogs move their heads (e.g., due to swallowing, respiration, heartbeat), which can have drastic effects on fMRI data. Especially if small brain regions like specific nuclei, which are much smaller than analogue areas in the human brain, are the focus of future investigation, movement by only a few millimeters is critical. Because these systematic artifacts contaminate the dog data more strongly than human data, particular emphasis should be placed on data cleaning (such as discarding scan volumes with excessive movement) and artifact removal methods. For instance, some researchers used an external infrared camera to track dog head motion and retrospectively correct for motion-related artifacts in the data. However, as some forms of motion cannot be corrected using standard motion-correction techniques, exploratory data analysis methods including temporal independent component analysis (ICA) and spin-history corrections are necessary. Many of these correction measures will require faster MR data sampling, such as in imaging sequences based on parallel imaging—which, as noted above, will require optimized head coils.

## Sample size

In contrast to both fMRI studies in humans and behavioral studies in dogs, the sample sizes of all published fMRI studies in dogs are small, never exceeding 13 dogs, resulting in strong outlier effects. This is unfortunate given the huge interindividual differences often obtained in cognitive dog research, possible differences between breeds (all studies used a mixture of breeds) and sex (spayed, neutered, intact). The time and effort required to train dogs for MRI, usually up to 4 months of training until a dog is ready for testing (Berns & Cook 2016), constitutes a major challenge for the future of fMRI in awake dogs.

## Task and stimulus design, experimental controls

Major challenges exist for fMRI approaches in dogs, as in humans, when it comes to linking neural responses to the cognitive processes they underpin. Issues such as reverse inference (i.e., drawing conclusions on cognitive processes based on brain activation only) are aggravated in dog fMRI, as the restrictive scanner environment precludes concomitant collection of cognitive or behavioral measures. Moreover, comparing human with canine brain responses requires closely matched stimuli and task designs. The future impact of canine fMRI will thus not only depend on overcoming technological and statistical challenges of the fMRI method but also on the creativity and rigor of the cognitive and behavioral scientists developing and implementing appropriate experimental designs and paradigms.
